# Incidental Detection of Asymptomatic Colonic Anisakiasis During Surveillance Colonoscopy: A Case Report and Literature Review

**DOI:** 10.1002/ccr3.72593

**Published:** 2026-04-24

**Authors:** Masashi Omori, Kenta Shibahara, Takashi Murakami, Eiji Kamba, Kei Nomura, Hirofumi Fukushima, Dai Ishikawa, Mariko Hojo, Tomoyoshi Shibuya, Akihito Nagahara

**Affiliations:** ^1^ Department of Gastroenterology Juntendo University Faculty of Medicine Tokyo Japan; ^2^ Innovative Microbiome Therapy Research Center Juntendo University Graduate School of Medicine Tokyo Japan; ^3^ Department of Pathophysiological Research and Therapeutics Gastrointestinal Diseases Juntendo University Graduate School of Medicine Tokyo Japan; ^4^ Department of Internal Medicine Juntendo University Koshigaya Hospital Saitama Japan

**Keywords:** anisakis sp., colonic anisakiasis, parasitic infection, surveillance colonoscopy

## Abstract

Anisakiasis is a parasitic infection caused by ingestion of raw or undercooked seafood containing Anisakis larvae. Although gastric involvement is common, colonic anisakiasis is rare and may be asymptomatic, making diagnosis difficult without endoscopic evaluation. We report a case of asymptomatic colonic anisakiasis incidentally detected during surveillance colonoscopy in a 53‐year‐old man 2 years after endoscopic submucosal dissection (ESD) for a sessile serrated lesion. The patient had consumed raw squid the evening before colonoscopy but had no abdominal symptoms. Bowel preparation was performed using an oral polyethylene glycol plus ascorbate solution. Colonoscopy revealed a worm‐like parasite embedded in the ESD scar in the cecum. The larva was successfully removed using biopsy forceps without complications. Histopathological examination confirmed anisakiasis, showing chronic inflammatory changes with focal neutrophilic infiltration. A review of previously reported cases suggests that colonic anisakiasis occurs predominantly in the right colon and is more frequently asymptomatic than generally recognized. Symptomatic cases typically present with abdominal pain and may occasionally lead to complications such as intussusception, whereas asymptomatic cases are often detected incidentally during endoscopy. This case highlights that colonic anisakiasis can be completely asymptomatic and incidentally identified, even after standard bowel preparation. Awareness of this condition and careful dietary history‐taking are important, particularly given the increasing global consumption of raw seafood, and endoscopic examination remains essential for accurate diagnosis and management.

## Key Clinical Message

Asymptomatic colonic anisakiasis is increasingly encountered with the widespread consumption of raw seafood. It may be incidentally detected during colonoscopy or mimic subepithelial lesions when larvae are not visible, underscoring the importance of careful endoscopic inspection and detailed dietary history for accurate diagnosis.

## 1 Introduction

Anisakiasis is a parasitic disease caused by the accidental ingestion of Anisakis larvae in raw or undercooked marine products. Infection occurs when third‐stage larvae penetrate the mucosa of the human gastrointestinal tract, producing a broad spectrum of clinical presentations ranging from transient abdominal discomfort to severe acute abdomen requiring emergency evaluation. Although the stomach is the most frequently affected site, colonic anisakiasis is considered exceedingly uncommon in clinical practice [1]. Cases diagnosed in the colon are often accompanied by acute abdominal pain, intestinal obstruction, or mass‐forming lesions that mimic neoplastic disease, whereas asymptomatic colonic involvement has been reported rarely.

Japan reports the largest number of anisakiasis cases worldwide because of the high consumption of raw seafood such as sashimi and sushi. However, the global incidence has also increased in recent decades [2, 3], likely reflecting diversification of dietary habits and the growing popularity of Japanese cuisine. This epidemiologic trend has drawn increasing clinical attention to atypical presentations, including involvement of the small and large intestine, in which diagnosis is often challenging because symptoms may be nonspecific or entirely absent [4]. Recognition of such cases is clinically important, as colonic anisakiasis can present as subepithelial lesions, inflammatory masses, or even intussusception, potentially leading to unnecessary surgical intervention [5, 6].

From a diagnostic standpoint, colonic anisakiasis is particularly difficult to identify before endoscopy. When symptoms are present, they overlap with those of other colonic disorders, including appendicitis, diverticulitis, inflammatory bowel disease, and gastrointestinal tumors. Moreover, imaging studies rarely identify larvae directly and typically demonstrate only nonspecific inflammatory changes [4]. Consequently, endoscopy remains the most reliable diagnostic modality, as it enables direct visualization and removal of larvae [6], followed by histopathologic confirmation.

The clinical manifestations of anisakiasis depend on both host immune responses and the extent of larval invasion [7]. Typical symptomatic cases develop acute abdominal pain within hours of ingestion due to mucosal penetration and localized eosinophilic inflammation. In contrast, asymptomatic or minimally symptomatic infections may occur when larval motility is limited or when immune activation is insufficient to trigger an IgE‐mediated immediate hypersensitivity response [8]. In such cases, detection is usually incidental during endoscopic examination and requires careful inspection, as larvae may be subtly embedded in the mucosa without causing marked edema or ulceration [6].

Here, we report a case of asymptomatic colonic anisakiasis incidentally detected during routine surveillance colonoscopy. The larva was found embedded at the site of a previous endoscopic submucosal dissection (ESD) in the cecum. In addition to presenting this case, we review previously reported cases of colonic anisakiasis, focusing on clinical differences between symptomatic and asymptomatic presentations. Through this combined approach, we aim to clarify the characteristics and diagnostic considerations of colonic anisakiasis, with particular emphasis on asymptomatic disease.

## 2 Case History/Examination

A 53‐year‐old man with a history of endoscopic submucosal dissection (ESD) for a sessile serrated lesion in the cecum underwent surveillance colonoscopy 2 years after the initial procedure. He had no notable medical history other than the prior ESD. He had consumed squid sushi on the evening before the examination. By the day of the procedure, he remained completely asymptomatic, reporting no abdominal pain, nausea, or diarrhea. On physical examination, his height was 170.6 cm and body weight was 74.2 kg. Vital signs were stable, with a body temperature of 36.2°C, blood pressure of 131/89 mmHg, heart rate of 72 beats per minute, respiratory rate of 14 breaths per minute, and oxygen saturation of 98% on room air. Bowel preparation was performed with 2 L of an oral polyethylene glycol plus ascorbate solution, starting 4 h before the procedure.

## 3 Investigations and Treatment

**FIGURE 1:**
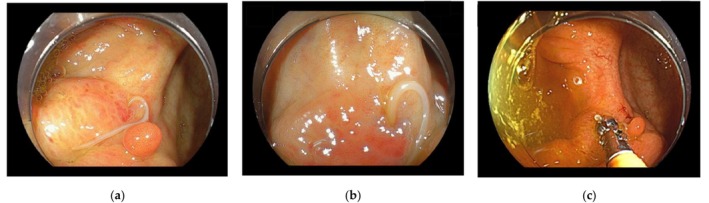
Endoscopic findings and removal of colonic anisakiasis at a prior ESD site. (a) A whitish, worm‐like Anisakis larva penetrating the colonic mucosa at the site of a previous endoscopic submucosal dissection near the appendiceal orifice. (b) Close‐up view showing mild erythema and localized swelling around the embedded larval head. No motility of the parasite body is observed. (c) Endoscopic removal of the embedded Anisakis larva using biopsy forceps. The larval head, together with a small portion of adherent mucosa, is grasped and successfully extracted without procedural complications. The removed specimen was subsequently submitted for histopathologic evaluation, confirming anisakiasis.

Colonoscopy was performed using an EC‐L600ZP7 endoscope (Fujifilm Corporation, Tokyo, Japan). A worm‐like parasite was identified at the previous ESD scar near the appendiceal orifice in the cecum (Figure 1a). The head of the parasite was embedded in the colonic mucosa and was associated with mild erythema and localized swelling (Figure 1b). No motility of the parasite body was observed. The embedded head was removed together with the adherent mucosa using biopsy forceps (Figure 1c), without any procedural complications.

## 4 Conclusion and Results

The specimen was submitted for pathological evaluation. Histopathological examination revealed colonic mucosa with moderate chronic inflammation and focal neutrophilic infiltration, along with morphological features characteristic of Anisakis. These findings confirmed the diagnosis of asymptomatic colonic anisakiasis.

After endoscopic removal of the larva, the patient remained asymptomatic, and no new symptoms developed during the follow‐up period.

## 5 Discussion

Anisakiasis of the digestive tract has been reported frequently in Japan, where consumption of raw seafood is common. In recent years, however, increasing consumption of raw seafood in Western countries has also led to a rising incidence of clinical anisakiasis [9, 10]. According to food poisoning surveillance data from the Ministry of Health, Labour and Welfare of Japan, 354 cases were reported in 2021, 578 cases in 2022, and 441 cases in 2023. Notably, in 2018, anisakiasis surpassed Campylobacter and norovirus infections to become the most common cause of food poisoning, accounting for 38.4% of reported cases [11]. In contrast, estimates based on medical claims data from 2011 to 2015, covering approximately 330,000 individuals, suggest a much higher annual incidence of approximately 7000 cases in Japan [12].

Gastrointestinal anisakiasis is generally classified into three types: gastric anisakiasis, intestinal anisakiasis, and ectopic anisakiasis. Among these, gastric anisakiasis is the most common form, accounting for 93.2% of reported cases, followed by involvement of the small intestine (2.6%). In contrast, cases involving the large intestine are rare, representing approximately 1.1% of reported cases [13]. Nevertheless, among cases of intestinal anisakiasis, asymptomatic infection has been reported to account for approximately 40% [14], a proportion higher than that observed in gastric anisakiasis, in which asymptomatic cases constitute approximately 20% [15]. Similarly, a report from seven Korean institutions described 20 cases of colonic anisakiasis diagnosed between 2002 and 2011, of which 8 cases (40%) were asymptomatic [16].

To further characterize colonic anisakiasis, we conducted a literature review using PubMed with the keyword “colonic anisakiasis,” covering the period from 1985 to 2025. After exclusion of two review articles, 34 case reports were identified [14, 17, 18, 19, 20, 21, 22, 23, 24, 25, 26, 27, 28, 29, 30, 31, 32, 33, 34, 35, 36, 37, 38, 39, 40, 41, 42, 43, 44, 45, 46, 47, 48]. Including the present case, a total of 35 cases of colonic anisakiasis were analyzed, and their clinical and endoscopic characteristics are summarized in Table 1. Among these 35 cases, 16 patients (46%) were asymptomatic, consistent with previous reports indicating that a substantial proportion of colonic anisakiasis cases may present without overt clinical symptoms.

**TABLE 1:** Clinical and endoscopic characteristics of reported cases of colonic anisakiasis, including the present case.

	Symptomatic, *n* = 19	Asymptomatic, *n* = 16	*p*
Age (year), mean ± SD (range)	53.4 ± 12.4 (24–70)	59.5 ± 10.6 (41–75)	0.19*
Sex			0.11**
Male	8 (42%)	11 (69%)	
Female	11 (58%)	5 (31%)	
Symptoms (Duplicates present)			
none	0 (0%)	16 (100%)	
abdominal pain	16 (84%)	0 (0%)	
Nausea・Vomiting	9 (47%)	0 (0%)	
hematochezia	3 (16%)	0 (0%)	
Location			
Cecum	1 (5%)	2 (13%)	
Ascending colon	8 (42%)	9 (56%)	
Transverse colon	5 (26%)	3 (19%)	
Descending colon	1 (5%)	1 (6%)	
Sigmoid colon	3 (16%)	0 (0%)	
Rectum	1 (5%)	1 (6%)	
Right colon	14 (74%)	14 (88%)	0.31**
Left colon	5 (26%)	2 (12%)	
Treatment			
Medication	2 (11%)	0 (0%)	
Endoscopic worm removal	9 (47%)	11 (69%)	
EMR・ESD	1 (5%)	3 (19%)	
Surgical operation	7 (37%)	2 (12%)	

*Note:* Summary of 35 cases of colonic anisakiasis reported in the literature between 1985 and 2025, including the current case.

* Mann–Whitney's U test.

** Chi‐square test.

The mean age of patients with symptomatic disease was 53.4 years, whereas that of asymptomatic patients was 59.5 years; this difference was not statistically significant (*p* = 0.19). With respect to sex distribution, the symptomatic group comprised 8 males and 11 females, while the asymptomatic group included 11 males and 5 females, with no statistically significant difference between groups (*p* = 0.11). Among symptomatic patients, more than 80% reported abdominal pain as the primary presenting symptom, followed by nausea and vomiting, reflecting the typical manifestations of acute gastrointestinal anisakiasis. Regarding lesion location, more than 70% of both symptomatic and asymptomatic cases involved Anisakis penetration in the right colon. No significant difference in lesion distribution was observed between the two groups (*p* = 0.31). Given the limited number of cases and the retrospective nature of this analysis, these statistical comparisons should be interpreted with caution.

Of the 19 symptomatic cases, 4 progressed to intussusception, and all of these patients required surgical intervention. In these cases, anisakiasis was confirmed either by pathological examination of resected specimens or by colonoscopic identification of Anisakis larvae at the leading edge of the invaginated bowel segment. In contrast, among asymptomatic cases, 11 larvae were incidentally identified and removed during endoscopic examination. However, 4 asymptomatic cases presented with a subepithelial lesion–like morphology that precluded an immediate diagnosis of anisakiasis. These lesions were subsequently resected by endoscopic mucosal resection, endoscopic submucosal dissection, or surgery, and pathological evaluation confirmed Anisakis infection in all cases.

Differentiating anisakiasis from true subepithelial lesions based solely on endoscopic appearance is often difficult; nevertheless, anisakiasis should be considered in the differential diagnosis when the lesion location and dietary history are suggestive. In this context, a detailed dietary history, particularly regarding recent consumption of raw seafood, remains essential. Although several reports [49] have described characteristic endoscopic ultrasound findings—namely, a prominent hyperechoic area within a hypoechoic mass extending from the third to fourth layers, with the hyperechoic component appearing disproportionately large relative to the surrounding hypoechoic lesion—establishing a definitive diagnosis based on endoscopic ultrasound alone remains challenging. When endoscopic ultrasound provides information regarding the layer of origin and suggests a benign process, and when procedural safety can be reasonably ensured, diagnostic endoscopic resection may be considered as a management option.

The pathogenesis of anisakiasis involves two principal mechanisms: (1) direct tissue invasion by larvae accompanied by localized inflammation, and (2) immune sensitization to Anisakis antigens leading to immediate‐type allergic reactions [50]. When larvae penetrate the gastric or intestinal wall, they cause tissue injury through mechanical disruption and the release of excretory–secretory products. This process activates the innate immune system, in which dendritic cells and macrophages phagocytose Anisakis antigens and subsequently promote differentiation of Th2 helper T cells. Th2 cytokines, including interleukin (IL)‐4, IL‐5, and IL‐13, then drive eosinophil proliferation and recruitment. Activated eosinophils release cytotoxic granules such as major basic protein and eosinophil cationic protein, which contribute to parasite elimination but simultaneously induce mucosal damage and inflammation [51]. Consequently, eosinophilic granulomas may form in the submucosa, resulting in mass formation, ulceration, or intestinal obstruction.

In parallel with local inflammatory responses, *Anisakis* possesses multiple allergenic molecules. The WHO/IUIS Allergen Nomenclature database currently lists 14 allergens (Ani s 1–14), including representative allergens such as Ani s 1, Ani s 2, Ani s 3, Ani s 7, and Ani s 13. In sensitized individuals, these antigens can induce specific IgE production, and subsequent exposure may activate mast cells and basophils, leading to immediate hypersensitivity reactions such as urticaria, asthma, and anaphylaxis [52]. Some *Anisakis*‐derived allergenic proteins have been reported to retain antigenicity after heating or freezing, suggesting that allergenic components may persist even after food processing; however, the clinical significance of such residual allergens remains controversial [53]. The immunological mechanisms underlying Anisakis‐induced allergy appear to be more complex than previously recognized.

Taken together, anisakiasis represents a complex immunopathological condition involving direct parasitic tissue injury, Th2‐mediated inflammation, and IgE‐dependent immediate hypersensitivity. Accordingly, its clinical manifestations range from localized gastrointestinal symptoms, such as abdominal pain and nausea, to systemic allergic reactions. A comprehensive understanding of these immunological mechanisms is therefore essential for improving diagnostic accuracy and guiding appropriate management strategies. In the present case, asymptomatic colonic anisakiasis was most likely caused by ingestion of raw squid on the day before colonoscopy. The absence of symptoms may be explained by a weak or delayed hypersensitivity response or by limited larval motility, which may have prevented deep mucosal penetration and subsequent IgE‐mediated reactions.

Given the global rise in raw seafood consumption, the incidence of anisakiasis is expected to continue increasing. Greater awareness of its diverse clinical presentations—including silent colonic involvement—is essential to avoid delayed diagnosis, unnecessary surgical interventions, and excessive treatment. In parallel, public health efforts promoting safe seafood handling and consumption practices will remain crucial in reducing the burden of anisakiasis worldwide.

In conclusion, asymptomatic anisakiasis represents a form of parasitic infection in which the ingestion of Anisakis larvae does not elicit overt clinical symptoms, making detection particularly challenging. While symptomatic anisakiasis typically presents with acute gastrointestinal complaints, asymptomatic cases often remain unrecognized unless incidentally identified during endoscopic examinations. Further research is needed to elucidate the immunologic and pathological mechanisms underlying asymptomatic infection and to clarify its broader public health implications.

## Author Contributions

**Masashi Omori:** conceptualization, investigation, writing – original draft. **Kenta Shibahara:** investigation, writing – review and editing. **Takashi Murakami:** conceptualization, project administration, writing – review and editing. **Eiji Kamba:** writing – review and editing. **Kei Nomura:** writing – review and editing. **Hirofumi Fukushima:** writing – review and editing. **Dai Ishikawa:** writing – review and editing. **Mariko Hojo:** writing – review and editing. **Tomoyoshi Shibuya:** writing – review and editing. **Akihito Nagahara:** conceptualization, supervision.

## Funding

The authors have nothing to report.

## Disclosure

Institutional Review Board Statement: This study was conducted in accordance with the Declaration of Helsinki. Because this was a single case report performed within the scope of routine clinical practice and did not involve interventional research involving human subjects, ethical review was waived.

## Consent

Written informed consent was obtained from the patient for the publication of this case report and any accompanying images. The authors confirm that written informed consent has been obtained from the patient.

## Conflicts of Interest

The authors declare no conflicts of interest.

## Data Availability

Data sharing is not applicable to this article because no new datasets were generated or analyzed beyond the information presented in this case report and the cited literature.
